# Motivating and inhibiting factors influencing the application of mhealth technology in post-abortion care: a review study

**DOI:** 10.1186/s12884-024-06253-0

**Published:** 2024-01-22

**Authors:** Zahra Zolfaqari, Haleh Ayatollahi, Fahimeh Ranjbar, Arezoo Abasi

**Affiliations:** 1 Department of Health Information Management, School of Health Management and Information Sciences, Iran University of Medical Sciences, Tehran, Iran; 2 Health Management and Economics Research Center, Health Management Research Institute, Iran University of Medical Sciences, Tehran, Iran; 3 Nursing Care Research Center, School of Nursing and Midwifery, Iran University of Medical Sciences, Tehran, Iran

**Keywords:** Abortion, Post-abortion care, Telehealth, Mhealth, Telemedicine

## Abstract

**Background:**

Abortion and its complications are challenges that endanger women's health, especially in developing countries. It seems that the application of mhealth technology can be useful as a safe and affordable strategy in post-abortion care. The purpose of this study was to identify factors influencing the use of mhealth technology in post-abortion care.

**Methods:**

This was a review study conducted in 2023 and articles published in English between 2010 and 1st November 2023 were searched in PubMed, Scopus, ProQuest, Web of Science, and Embase databases as well as Google Scholar. Data were collected using a data extraction form and were analyzed narratively.

**Results:**

The influencing factors could be divided into the motivating and inhibiting factors. The motivating factors included the subgroups of the individual factors (e.g., saving time), technical factors (e.g., usability), economic factors (e.g., saving costs), and ethico-legal factors (e.g., improving security and confidentiality of the information). Similarly, the inhibiting factors consisted of individual factors (e.g., fear of expressing abortion), technical factors (e.g., a lack of access to the network and the Internet), economic factors (e.g., inappropriate patient financial status), and ethico-legal (e.g., concerns over the security and confidentiality of information).

**Conclusion:**

This study underscores the importance of considering various technical and non-technical factors influencing the design and implementation of mHealth technology in post-abortion care. Developers need to address these factors to ensure successful technology adoption and mitigate risks. The findings contribute to the enhancement of women's health and offer insights for future technology acceptance models in the mHealth field.

## Introduction

Abortion is one of the most common causes of pregnancy termination and fetal loss. According to the World Health Organization (WHO), in terms of safety, abortion can be divided into safe, less safe, and unsafe abortion, and in terms of the method of abortion, it can be categorized as induced, medical, and surgical abortion. The statistics of the last 50 years show that the ratio of unsafe abortions to total abortions is up to 31% in countries with prohibiting abortion law and it is up to 49% in developing countries [1].

Generally, abortion is a stressful and dangerous event for women [2]. These women may experience lots of issues, such as pain management, infection, medication use, bleeding, long-term complications, wound management, depression, challenges of getting pregnant again, and the need for future healthcare interventions [3]. Therefore, providing post-abortion care to minimize complications and women's deaths, preventing incomplete abortions, treating complications, and reducing the number of unwanted pregnancies along with providing healthcare recommendations seem necessary [4].

It should be noted that improving women's health is one of the main priorities of the health system in each country. In this regard, women's access to healthcare information, services, and post-abortion care is not only considered as a human right but also is regarded as a sign of national development [3–8]. Despite the women’s need to receive post-abortion care, usually due to the high costs of the care or the distance from health care centers, receiving post-abortion care is interrupted resulting in an increase in the rate of illnesses and mortality in these people [9]. Four out of every 10 women who experience an abortion require healthcare services for acute and severe complications and three percent of them are at risk of death [10, 11]. Therefore, to reduce the consequences of high-risk pregnancy and preserve the health status of women and babies, the use of telemedicine services has been suggested [12, 13]. ​​Telemedicine comprises a wide range of technologies, including m-health [14], which refers to the use of portable wireless devices that are capable of transmitting, storing, processing, and retrieving data as well as connecting patients and healthcare providers [15]. Five types of mhealth devices include smartphone-based applications, smartphone-connected devices, wearable and wireless devices, handheld-imaging platforms, and miniaturized sensor-based technologies [16].

MHealth technologies exhibit substantial promise in advancing post-abortion care by augmenting healthcare accessibility, efficiency, and patient outcomes [17]. In post-abortion care, it goes beyond traditional healthcare settings, using mobile applications to provide essential information, support, and follow-up resources. This integration offers a chance to overcome healthcare barriers, especially in resource-limited regions, where comprehensive and timely access to care might be limited. Through the use of mHealth applications, individuals can bridge gaps in healthcare accessibility, promoting a patient-centric and continuous approach to post-abortion care [18, 19]. Using mhealth technology, healthcare providers can use text messages, voice messages, and video calls to be in touch with patients [20], and patients can access medical information related to their condition at any time and place and can receive their required care [21]. However, there are a range of considerations, including socio-economic disparities, cultural nuances, and variations in technological literacy, which can significantly impact the adoption and efficacy of mHealth solutions in diverse populations [22].

Some studies showed that these technologies can be used for safe abortion and post-abortion care, while significantly reducing the complications of abortion [23]. Healthcare providers also tend to use these technologies to provide many other services [24, 25].

This research tackles the persistent challenge of unsafe abortions and associated complications, particularly prevalent in developing countries, contributing to a staggering 55% of maternal mortality [26]. Despite decades since the World Health Organization recognized this issue, unsafe abortion continues to impose severe health burdens [1].

To encourage women to use different types of health information technology, especially in a context that social barriers are serious, several technical and non-technical factors should be considered. For instance, factors such as ease of use, acceptability, individuals’ willingness to use the technology, information confidentiality, reliability, and appropriate design of mhealth technology play a vital role in the acceptance and use of this technology [24, 25, 27–33]. It seems that identifying these factors may facilitate the use of mhealth technology in post-abortion care and will help to be more successful in preventing abortion complications, monitoring women’s quality of life, and providing solutions for possible obstacles to the implementation of future systems. This study aimed to elucidate the pivotal factors influencing the adoption of mHealth technology in post-abortion care, presenting contextual insights derived from diverse settings. By proactively addressing identified inhibiting factors, our goal is to provide actionable solutions that can inform policymakers and healthcare providers. This contribution is vital for harnessing the transformative potential of mHealth in enhancing women's access to high quality healthcare, particularly in developing countries. Through a systematic examination of motivating and inhibiting factors, we strive to empower healthcare providers, policymakers, and technology developers with the knowledge essential for crafting effective and patient-centric mHealth solutions tailored for post-abortion care.

The study underscores the urgency of innovative solutions aligned with Sustainable Development Goals to improve post-abortion care. Focusing on the current underutilization of mHealth technology in this context, our research seeks to unveil factors influencing its acceptance and use [34]. By identifying these factors, the study endeavors to pave the way for the effective implementation of mHealth, bridging critical gaps in women's healthcare and the delivery of post-abortion care [35]. The overarching objective is to influence positive outcomes by promoting the widespread and impactful integration of mHealth solutions in post-abortion care.

## Methods

This was a review study carried out in 2023. Before conducting the research, ethics approval was obtained from the National Ethics Committee of Biomedical Research (IR.IUMS.REC.1399.596).

### Search strategy

Articles related to the factors influencing the use of mhealth technology in post-abortion care were searched in PubMed, Scopus, Web of Science, ProQuest, Embase databases and Google Scholar. The search strategy used in PubMed was as follows:

- (("Abortion"[Title/abstract] OR "induced abortion"[ MeSH Terms] OR "Post-abortion care"[Title/abstract] OR "Medical abortion"[Title/abstract] OR "Pregnancy monitoring"[Title/abstract] OR "unsafe abortions"[Title/abstract] OR "Legal abortion"[ MeSH Terms] OR "criminal abortion"[ MeSH Terms] OR "pregnancy prevention"[Title/abstract] OR "Post abortion"[Title/abstract] OR "abortion Complications"[Title/abstract]) AND ( "eHealth"[Title/abstract] OR "mHealth"[Title/abstract] OR "personal health records"[Title/abstract] OR "digital health"[Title/abstract] OR"telemedicine"[ MeSH Terms] OR "telehealth"[Title/abstract] OR "Health informatics"[Title/abstract]))

### Inclusion and exclusion criteria

This study included English-language articles on mHealth in post-abortion care published from 2010 to 1st November 2023. Exclusions comprised of book chapters, letters, and commentaries, non-English articles, those lacking full texts, and those diverging from the study aim which was identifying factors influencing the use of mhealth technology in post-abortion care. In fact, articles not primarily emphasizing mHealth services for post-abortion care, and those focusing on unrelated issues such as using telemedicine for medical abortion were excluded.

### Data analysis

Adhering to the Preferred Reporting Items for Systematic Reviews and Meta-Analysis (PRISMA) flow diagram [36], our screening process was systematically implemented. Upon obtaining relevant articles, EndNote software (X20, Clarivate) was employed for meticulous reference management, ensuring the elimination of duplicates. After removing duplicates, the title and abstract of the remaining articles were reviewed.

A data extraction form was used to determine factors influencing the use of mhealth technology in post-abortion care. This form included the name of the authors, year of the study, name of the country, research objective, type of study, and factors influencing the use of mhealth technology in post-abortion care. To report the findings, factors influencing the use of mhealth technology in post-abortion care were initially divided into two categories, motivating and inhibiting factors. Then, using the method of content analysis, the results were extracted, tabulated, summarized, and finally synthesized narratively.

### Classification of influencing factors

The factors shaping the acceptance and adoption of mhealth technology in post-abortion care were thoughtfully organized into key domains, showcasing a nuanced understanding. The factors influencing the acceptance and utilization of mhealth technology for post-abortion care were broadly categorized into individual, technical, economic, and ethico-legal domains. Individual factors included several items such as willingness to receive counseling, and educational level. Technical factors were related to the technological aspects of mhealth technology implementation, including multilingual support, readability of messages, customization options, accessibility challenges, and usability issues. Economic factors delve into the financial aspects, addressing cost reduction, affordability for patients, and the ability to compare costs for different services. Lastly, ethico-legal factors considered the ethical and legal implications, such as data security, concerns about privacy, and the availability of websites or apps to the general public. This comprehensive framework provided a structured understanding of diverse factors influencing the integration of mhealth technology in post-abortion care.

## Results

**Fig. 1:**
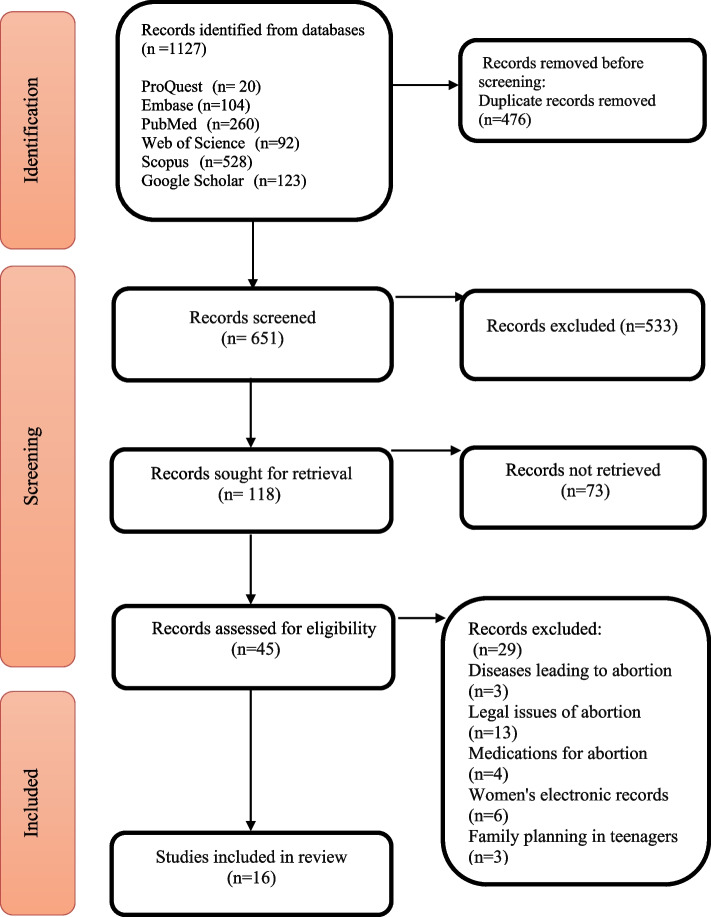
PRISMA flow diagram [37]

Initially, 1127 articles were retrieved by searching databases, and 476 duplicate articles were excluded. The remaining articles (*n* = 651) were examined in terms of the title and abstract relevancy to the research topic. After excluding 532 irrelevant articles, the full texts of 46 articles were studied. Finally, 16 studies related to the research topic were selected and included in the research. The screening process of the articles was presented in the Preferred Reporting Items for PRISMA flow diagram (Fig. 1).

According to the results, the articles included in the current research (*n* = 16) were conducted in Canada [19, 30, 35], Kenya [38], Venezuela [39], Vietnam [40], Pakistan [41], Myanmar [42], Cambodia [20, 43–46], Australia [47], Bangladesh [17], and South Africa [48] between 2014 and 2023. Among them, 13 articles (81.2%) discussed both motivating and inhibiting factors and three studies focused on motivating factors. A summary of each study, including the name of the authors, year of the study, name of the country, research objective, research methodology, and factors influencing the use of mhealth technology in post-abortion care was presented in Table 1.

**Table 1:** Summary of the selected studies

NO	Authors	Country	Objective	Type of Study	Factors influencing the use of mhealth technology in post-abortion care		
1	Luigi-Bravo et al. 2023 [19]	Canada	Sharing insights and lessons from implementing three mobile health interventions for self-managed abortion and reproductive healthcare	Qualitative (Literature review)	Motivating factors	Ethico-legal	Information security and confidentiality
						Individual	Receiving information about the complications of abortion and methods of preventing re-pregnancy Ease of access to care from anywhere at anytime
						Technical	Usability and comprehensiveness
					Inhibiting factors	Ethico-legal	Legal and cultural barriers Stigma and discrimination
						Individual	Patient’s low level of education Lack of mobile phone ownership by the patient
						Technical	Problems with the mobile network or the Internet in the deprived areas
2	Ngo et al. 2023 [40]	Vietnam	Assessing the impact of a mHealth app, specifically the iConnect app, on improving knowledge, attitudes, and behaviors related to safe abortion among female sex workers (FSWs) in Hanoi Evaluate the feasibility, acceptability, and effectiveness of the mHealth intervention in addressing the high risk of unsafe abortions	Quantitative (Pre- post test)	Motivating factors	Individual	Receiving information about the complications of abortion and methods of preventing re-pregnancy Ease of access to care from anywhere at anytime
						Technical	Ease of receiving information via mobile phones
					Inhibiting factors	Individual	Patient’s low level of education
						Technical	Usability issues and scattered information Problems with the mobile network or the Internet in the deprived areas
3	Luigi-Bravo et al. 2022 [39]	Venezuela	Explore the potential role of mHealth in the context of abortion, specifically focusing on self-managed medical abortion in a complex humanitarian emergency	Qualitative (Literature review)	Motivating factors	Individual	Receiving information about the complications of abortion and prevention of re-pregnancy Receiving emotional and informational support from the professional staff
					Inhibiting factors	Ethico-legal	Legal and sociopolitical constraints
						Individual	Receiving inadequate emotional support
						Technical	Usability issues and scattered information Problems with the mobile network or the Internet in the deprived areas
						Economic	Inappropriate patient financial status
4	Sudhinarase et al. 2022 [38]	Kenya	Investigating the impact of a person-centered abortion care mobile intervention, delivered by either peer counselors or nurses through mobile phones, on mental health, social support, and abortion-related stigma among Kenyan women post-abortion in private clinics Comparing outcomes with standard care emphasizes the growing role of mHealth in addressing sensitive healthcare issues in restrictive settings	Quantitative (randomized controlled trial)	Motivating factors	Ethico-legal	Information security and confidentiality
						Individual	Receiving information about the complications of abortion and methods of preventing re-pregnancy Interactive communication with the professional staff Receiving emotional and informational support from the professional staff Ease of access to care from anywhere at anytime
					Inhibiting factors	Ethico-legal	Legal and Cultural barriers
						Individual	Receiving inadequate emotional support
5	Shaikh et al. 2021 [41]	Pakistan	Evaluate the implementation of a hybrid telemedicine-community accompaniment model for sexual and reproductive health services, with a focus on abortion and contraception	Quantitative (survey)	Motivating factors	Ethico-legal	Information security and confidentiality
						Individual	Receiving information about the complications of abortion and methods of preventing re-pregnancy Interactive communication with the professional staff Ease of access to care from anywhere at anytime
					Inhibiting factors	Individual	Challenges with communication via the app Fear of talking about abortion
						Technical	Problems with the mobile network or the Internet in the deprived areas
6	Aung et al. 2020 [42]	Myanmar	Evaluating the effectiveness of mhealth interventions for preventing re-pregnancy Identifying mhealth features and behavior change communication components used in the interventions	Qualitative (Systematic review)	Motivating factors	Ethico-legal	Information security and confidentiality
						Individual	Receiving information about the complications of abortion and methods of preventing re-pregnancy Interactive communication with the professional staff Receiving emotional and informational support from the professional staff Ease of access to care from anywhere at anytime Mobile phone ownership Saving time
						Economic	Saving costs
					Inhibiting factors	Ethico-legal	Concerns over information security and confidentiality
						Individual	Concerns over incorrect and invalid information Lack of mobile phone ownership by the patient
7	Hill et al. 2020 [46]	Cambodia	Evaluating the cost-effectiveness of mobile phone-based support for post-abortion family planning in Cambodia	Quantitative (cost-effectiveness analysis)	Motivating factors	Individual	Receiving unique support for each patient Ease of access to care from anywhere at anytime
						Economic	Saving costs Inexpensive health care services compared to the face-to-face visits
8	Ireland et al. 2020 [47]	Australia	Exploring and better understanding of women’s access to teleabortion in rural areas of Australia	Qualitative (structured interview)	Motivating factors	Individual	Interactive communication with the professional staff Feeling of not being judged Feeling of comfort when communicating via the Internet Saving time
						Economic	Inexpensive and cost-effective services
					Inhibiting factors	Individual	Patient’s low level of education Lack of self-confidence Fear of talking about abortion
9	Biswas et al. 2020 [17]	Bangladesh	Assessing the feasibility and acceptance of SMS-based m-health interventions in post-abortion pregnancy prevention in Bangladesh	Randomized controlled trial	Motivating factors	Individual	Patient’s high level of education
						Economic	Patient financial status
						Technical	Multilingual support Readability of messages Setting time for receiving messages
					Inhibiting factors	Individual	Patient’s low level of education Lack of mobile phone ownership by the patient
						Economic	Inappropriate patient financial status
10	Gill et al. 2019 [30]	Canada	Examining how women use mobile phones to access health care information and their preferences for the content and design of mhealth interventions	Mixed- methods study (quantitative–qualitative)	Motivating factors	Ethico-legal	Information security and confidentiality
						Individual	No feeling of embarrassment when expressing abortion Receiving emotional and informational support from the professional staff Withdrawing the intervention whenever they want
						Technical	Ease of receiving information via mobile phones Ease of using mobile phones Usability and comprehensiveness
						Economic	Saving cost
11	Gill et al. 2019 [35]	Canada	Assessing the usability of a website intervention to determine its usability and appropriateness	Quantitative	Motivating factors	Ethico-legal	Registering on the websites and passing identity check
						Individual	Access to up-to-date and comprehensive information about all types of abortion
						Technical	Usability Understandable terminology Usability of the websites
						Economic	Comparing costs for different types of services
					Inhibiting factors	Ethical-legal	Concerns over lack of privacy Unavailability of the website to the public
						Individual	Receiving inadequate emotional support
						Technical	Usability issues and scattered information
12	Smith et al. 2017 [45]	Cambodia	Evaluating patients' interaction with the interventions of Mobile Technology for Improved Family Planning (MOTIF) from the service provider's perspective	Quantitative	Motivating factors	Individual	Receiving information about the complications of abortion and prevention of re-pregnancy Ease of access to care from anywhere at anytime
					Inhibiting factors	Individual	Patient’s low level of education Lack of time to use mhealth technology Lack of mobile phone ownership by the patient
						Technical	Problems with the mobile network or the Internet in the deprived areas
						Economic	Inappropriate patient financial status
13	Smith et al., 2017 [43]	Cambodia	Examining the views and experiences of women participating in the interventions provided through Mobile Technology for Improved Family Planning,	Qualitative	Motivating factors	Individual	Receiving information about the complications of abortion and prevention of re-pregnancy Receiving emotional and informational support from the professional staff Saving time Ease of access to care from anywhere at anytime
						Technical	Ease of receiving information via mobile phones
						Economic	Saving costs
					Inhibiting factors	Ethico-legal	Concerns over lack of privacy
						Technical	Inappropriate number and time of receiving messages via mobile phone
14	Smith et al. 2016 [20]	Cambodia	Providing an overview of the formative research process used in a mobile phone intervention for post-abortion re-pregnancy prevention	Mixed-methods study	Motivating factors	Individual	Receiving information about the complications of abortion and prevention of re-pregnancy Patient’s high level of education Saving time
						Technical	Ease of receiving information via mobile phones
						Economic	Saving costs Patient financial status
					Inhibiting factors	Ethical-legal	Concerns over lack of privacy
						Individual	Lack of mobile phone ownership by the patient
						Technical	Lack of multilingual support
15	Smith et al. 2015 [44]	Cambodia	Evaluating the effectiveness of a mobile phone-based intervention in preventing pregnancy after abortion in Cambodia	Quantitative (randomized trial)	Motivating factors	Individual	Receiving unique support for each patient Ease of access to care from anywhere at anytime
						Technical	Ease of receiving information via mobile phones
						Economic	Inexpensive and cost-effective services compared to the face-to-face visits
16	De Tolly et al. 2014 [48]	South Africa	Assessing the feasibility and efficiency of information and follow-up provided by mobile phone after medical abortion	Quantitative (randomized controlled trial)	Motivating factors	Individual	Receiving information about the complications of abortion and prevention of re-pregnancy Receiving emotional and informational support from the professional staff Ease of access to care from anywhere at anytime
						Technical	Ease of receiving information via SMS Customizing programs Multilingual support
					Inhibiting factors	Ethico-legal	Concerns over lack of privacy
						Individual	Concerns over incorrect and invalid information Lack of mobile phone ownership by the patient Remembering difficulties of abortion
						Technical	Problems with the mobile network or the Internet Receiving duplicate information

### Motivating factors influencing the use of mhealth technology in post-abortion care

Motivating factors influencing the use of mhealth technology in post-abortion care were mentioned in all selected studies. The objectives of these studies were mainly related to the use of mhealth in preventing pregnancy after an abortion [17, 19, 39–45], the feasibility of using mhealth technology for post-abortion care [30, 40, 47, 48], the cost-effectiveness of mhealth interventions after an abortion [46] and the use of mhealth in post-abortion family planning [20]. The analysis of the motivating and inhibiting factors across the diverse mHealth intervention studies reveals key insights into the adoption and success of these interventions.

Motivating factors included individual, ethico-legal, economic, and technical factors, which are described in the following sections.

#### Individual factors

These factors were discussed in all selected studies (*n* = 16, 100%). Aung et al. conducted a systematic review to show the impact of mhealth interventions on pregnancy prevention after abortion. They found that the individual motivating factors included the desire to receive information about the complications of abortion and methods of preventing pregnancy after abortion, interactive communication with the professional staff, receiving emotional and informational support from the professional staff, mobile phone ownership, and saving time [42]. Similarly, Smith et al. highlighted these factors in different studies [20, 43–45]. In Hill et al.’s study, providing unique support for each patient and addressing her problems were among the individual motivating factors [46]. Ireland et al. found that factors, such as the feeling of not being judged, the feeling of comfort when communicating via the Internet, and saving time motivated people to use mhealth technology [47]. According to Biswas et al., patients’ level of education is also influenced by using or not using the technology [17].

Gill et al. noted that patients have no feeling of embarrassment when expressing abortion problems in virtual care. As a result, they can receive appropriate emotional and informational support from the professional staff. Moreover, they can stop using the technology or withdraw from the intervention whenever they want [30]. In another study, Gill et al. stated that getting access to up-to-date and comprehensive information about different types of abortion is another individual motivating factor for using mhealth technology[30]. These factors were also highlighted in De Tolly et al.’s study [48].

Luigi-Bravo et al. identified individual motivating factors such as increased access to information and easy access to accurate and timely information [19]. In two studies, an increased accessibility to post-abortion support, overcoming geographical barriers, and immediate post-abortion relief were reported [38, 39]. The easy access to information, privacy and confidentiality and risk mitigation were reported in other studies [40, 41].

#### Ethico-legal factors

Seven out of 16 papers (43.7%) highlighted the role of ethico-legal factors. Gill et al. and Aung et al. emphasized the role of information security and confidentiality as one of the motivating factors for using mhealth technology [30, 42]. According to Gill et al., patients preferred using applications or health websites that needed registration, and asked them to pass identity checks when using the applications [30]. Similarly, other studies discussed the role of ethico-legal factors in using mhealth technology in post-abortion care [38, 39, 41].

#### Economic factors

According to different researchers, saving costs was one of the advantages of using mhealth technology which could also motivate people to use it [30, 42, 43, 46]. Similarly, Ireland et al. highlighted the role of the technology cost-effectiveness in increasing its usage [47]. In two studies, the researchers reported that patient’s financial status may influence using the technology [17, 20]. Another motivating factor was related to the possibility of the comparing cost of teleconsultation and face-to-face consultation which showed that the first one was cheaper [30, 44].

#### Technical factors

In 2020, Biswas et al. conducted a randomized controlled trial to investigate the feasibility and acceptance of SMS-based mhealth interventions in post-abortion pregnancy prevention. In their study, multilingual support, the readability of messages, and setting time for receiving messages were mentioned as technical motivating factors [17]. In two studies, ease of receiving information through mobile phones, ease of using mobile phones, usability and comprehensiveness were found as important technical factors [30, 48]. Luigi-Bravo et al. emphasized the importance of a user-centered design in the implementation of mHealth interventions for post-abortion care [19]. Similarly, in the study conducted by Ngo et al., the significance of receiving automated educational messages for effective post-abortion care was highlighted [40]. Other technical factors included simple layout and design of the websites, simple and understandable terminology, ease of information retrieval, usability of the websites, possibility of customizing messages, and the correct functioning of the links [30, 43].

### Inhibiting factors influencing the use of mhealth technology in post-abortion care

Inhibiting factors affecting the use of mhealth technology in post-abortion care were stated in 13 studies (81.2%). These studies were conducted in Canada [19, 35], Kenya [38], Venezuela [39], Vietnam [40], Pakistan[41], Myanmar [42], Cambodia [20, 43, 45], Australia [47], Bangladesh [17], and South Africa [48] between 2014 and 2023. The inhibiting factors included individual, ethico-legal, economic, and technical factors which are described in the following section.

#### Individual factors

Aung et al. argued that despite the patients' willingness to use mhealth technology, concerns over the validity of the information and the lack of mobile phone ownership were among the factors preventing patients from using mhealth technology in post-abortion care [42]. Ireland et al. noted that patients' low level of education, lack of self-confidence, and fear of talking about abortion were other inhibiting factors [47]. These factors along with challenges related to communication via the app [19, 39–41], receiving inadequate emotional support [38], and the lack of time to use mhealth technology [45] were also highlighted by other researchers..

#### Ethico-legal factors

Some patients were concerned about the explaining their conditions, confidentiality issues, and privacy of information when using mhealth technology in post-abortion care [19, 20, 35, 38, 39, 42, 43, 48]. Biswas et al. added that the availability of health websites for the public without maintaining security and privacy issues is another ethico-legal inhibiting factor [17].

#### Economic factors

In terms of the economic factors, as mentioned in three studies, inappropriate patient's financial status was considered an inhibiting factor [17, 39, 45], as patients may not be able to pay for mhealth services.

#### Technical factors

Technical challenges, such as infrastructure limitations and design complexity, pose significant hurdles [17, 19, 20, 35, 39–41, 43, 45, 48]. Poor Internet speed and network connectivity, usability issues and scattered information, lack of multilingual support [20], and receiving duplicate information [48] may also hinder the effectiveness of mHealth interventions.

### Synthesis

In general, despite numerous successful instances of implementing telemedicine and mHealth in post abortion care, the adoption of technology can still be influenced by various factors. These factors are nuanced and depend on the patient's specific condition, priorities, perceptions, and the intended purposes of utilizing the technology. Within the scope of this study, a range of motivating and inhibiting factors were identified, shedding light on the complexities of mHealth implementation in post-abortion care. Recognizing and understanding these factors is pivotal, as leveraging motivating elements enhances the likelihood of successful technology implementation, while overlooking them may result in implementation failure. Therefore, a thorough identification and detailed consideration of these factors are essential to effectively meet the diverse requirements of users in the context of post-abortion care.

## Discussion

### Principle findings

The purpose of this study was to identify factors influencing the use of mhealth technology in post-abortion care. The review of selected articles indicated that these factors can be divided into two categories, namely, motivating factors and inhibiting factors. These factors included some individual, technical, economic, and ethico-legal factors, too.

According to the literature, the use of mhealth technology can help improve quality of care and service delivery, as well as reducing human errors and the cost of care [49]. Despite the potentials of this technology to provide services at any time and place, the acceptance and continuous use of it by the end-users are among the most important concerns of the system developers, as the use of mhealth technology does not necessarily mean its acceptance [50]. Gill et al. suggested that patient-centered design is a suitable and useful method for planning and carrying out interventions for patients, especially in post-abortion care [30].

In the study conducted by Mutua et al., motivating and inhibiting factors influencing the implementation of telemedicine included the legal factors (e.g., liability and jurisdiction, clinical governance, informed consent, data confidentiality, and security), cultural factors (e.g., language, trust, and resistance to change), contextual factors (e.g., resources and infrastructure), and sustainability factors (e.g., cost, integration into the national health systems, and financing) [4]. Regarding the use of mhealth in post-abortion care, Aung et al. indicated that individual motivating factors include patient's need to receive information about the abortion complications and methods of preventing pregnancy after abortion, communicate with the clinical staff to receive appropriate emotional and informational support, access to get care at any time and place at a low cost [42]. In other studies, providing unique clinical and emotional support for each patient and addressing her problems were among the individual motivating factors [46, 51]. While in the current study, motivating and inhibiting factors were identified in detail, some researchers used other approaches to categorize influencing factors. For example, Zhang et al. used the technology acceptance model (TAM) and reported that perceived ease of use and perceived usefulness are significant reasons for the acceptance and use of mhealth technologies [52]. Similarly, Mohamed et al. found that in some cases, perceived usefulness is more important than perceived ease of use for users [29]. Grossman et al. mentioned that ease of access to health care services at any time and place is one of the factors influencing the use of mhealth technology, and the effectiveness of these services are comparable with face-to-face clinical visits [53]. Zahedul-Alam et al. showed that performance expectancy, effort expectancy, social influence, facilitating conditions, and perceived reliability have a significant impact on the willingness to use mhealth technology [54]. It should be noted that in the present study, although the technology acceptance models and their variables were not examined, the research findings are generally consistent with the findings of other similar studies.

In terms of inhibiting factors, our study resonates with the findings of several studies in which network connection problems, lack of internet access in deprived areas, and inappropriate system design were commonly reported as technical inhibiting factors [19, 20, 35, 39, 41, 43, 45, 48]. Ngo et al., reported insufficient facilities, equipment, budget constraints, and clinicians' knowledge gaps as inhibiting factors [40].

Some studies investigated users’ experiences of using telemedicine technology. In this study, insufficient facilities, equipment, budget, and knowledge of clinicians about using the technology were reported as other factors inhibiting the use of telemedicine [55]. In addition, one of the main concerns of the users in using mhealth technology is the privacy issues [48]. Therefore, data protection and privacy have been recognized as the most essential features required for developing mhealth systems [56].

Another factor that can negatively influence the acceptance and use of mhealth technology is resistance to change, which can be caused by the lack of awareness of the usefulness of the technology [25]. In some cases, the resistance might be due to some cultural beliefs ​​and traditions, which can be resolved through education [57]. It seems that improving financial, organizational, and technical support along with creating changes in the thoughts, feelings, and attitudes of users would help increase the acceptance of new technologies [28]. Given the sensitivity of healthcare, especially in terms of patient health conditions, technology acceptance issues must be meticulously considered in the early stages of system design and implementation, as emphasized in a study by Luigi-Bravo et al. [19, 39, 52].

It is notable that the exploration of motivating and inhibiting factors in diverse mHealth interventions provides crucial insights for developing patient-centered systems. Prioritizing patient needs is essential for fostering engagement, trust, and positive health outcomes. Designing patient-centered mHealth systems requires a multifaceted approach, understanding individual preferences and many other factors for personalized support.

### Research limitations

The current study has certain limitations. Firstly, despite conducting a thorough search across selected databases, it is possible that some relevant studies were inadvertently omitted from the review. The vast and dynamic nature of research literature makes it challenging to ensure absolute inclusivity. Secondly, the inclusion criterion for articles written exclusively in English might introduce a language bias, potentially overlooking pertinent studies in other languages. The exclusion of studies in languages other than English may have limited the scope of our review, and there could be valuable insights from non-English literature that were not incorporated. Future research endeavors should aim for broader language inclusivity to enhance the comprehensiveness and representativeness of the findings.

## Conclusion

In conclusion, the findings of this review shed light on the critical motivating and inhibiting factors influencing the application of mHealth technology in post-abortion care. Understanding these factors is essential in designing and implementing different types of health information technologies including health. The identified factors, spanning individual, ethico-legal, economic, and technical dimensions, underscore the multifaceted nature of challenges and opportunities in leveraging technology for women's health. The motivating factors, such as the desire for information, saving time, cost-effectiveness, improved healthcare access, and enhanced information security; emphasize the potential benefits of mHealth adoption. These factors not only address practical aspects, but also highlight the importance of user-centred design and a patient-centered approach in developing mHealth solutions. Conversely, inhibiting factors like fear of expressing abortion, privacy concerns, financial limitations, and technical challenges highlight the need for nuanced strategies to overcome barriers, ensuring trust, data security, and inclusive mHealth interventions. Collaboration between system developers and healthcare policymakers is crucial for seamless integration into existing healthcare systems. Recognizing the interplay between technical and non-technical factors opens avenues to prevent abortion complications and revolutionize post-abortion care. These lessons provide valuable guidance for policymakers and service providers, fostering a deeper understanding of how to enhance community health, particularly for women. Looking ahead, exploring various models of technology acceptance in the mHealth field, and potentially developing new models for specific health conditions, holds promise. It is crucial to perceive mHealth technology not merely as a tool, but as a catalyst for positive change in post-abortion care. Taking proactive measures to enhance user experience, address inhibiting factors, and uphold ethical and secure technology use will be pivotal in unlocking the full potential of mHealth, ensuring the well-being of women's health.

## Data Availability

The datasets used and/or analyzed during the current study are available from the corresponding author upon a reasonable request.

## Abbreviations

WHO: World Health Organization
PRISMA: Preferred Reporting Items for Systematic Reviews and Meta-Analyses
TAM: Technology Acceptance Model
